# Direct gaze facilitates rapid orienting to faces: Evidence from express saccades and saccadic potentials

**DOI:** 10.1016/j.biopsycho.2016.10.003

**Published:** 2016-12

**Authors:** Inês Mares, Marie L. Smith, Mark H. Johnson, Atsushi Senju

**Affiliations:** a Centre for Brain and Cognitive Development, Department of Psychological Sciences, Birkbeck, University of London, Henry Wellcome Building, Malet Street, London WC1E 7HX, United Kingdom; b Department of Psychological Sciences, Birkbeck, University of London, Malet Street, London WC1E 7HX, United Kingdom

**Keywords:** Saccade-locked event-related potentials, Express saccades, Direct gaze, Face detection

## Abstract

Direct gaze is a crucial signal in human social communication, which is known to attract visual attention and modulate a wide range of behaviours. The present study investigated whether direct gaze facilitates rapid orienting to faces, which is important for adaptive on-line communication, and its neural correlates. Fifteen participants performed a rapid orienting task, in which they were instructed to saccade to peripherally presented buildings or faces containing direct or averted gaze as quickly as possible. Electroencephalographic recordings were made during the task. Shorter express saccade latencies were found for faces with direct gaze, compared to averted gaze or buildings, while no significant difference was found between faces with averted gaze and buildings. Furthermore, saccade-locked event-related potential (ERP) amplitudes in parieto-occipital areas discriminated faces with direct gaze from buildings and faces with averted gaze corroborating behavioural results. These results show that detection of direct gaze facilitates rapid orienting to faces.

## Highlights

• We investigated the role of direct gaze for rapid orienting to faces.
• Faster express saccades towards faces occurred only when faces were with direct gaze.
• Saccade-locked ERPs discriminated between gaze directions from the saccade onset.

## 1 Introduction

One of the hallmarks of social communication is its dynamic and rapidly changing nature. Successful social communication often relies on the immediate detection of social cues and a timely response. For example, missing a quick glance from a social partner can hinder successful communication. Thus, fast detection and orientation to social and communicative signals is crucial for social adaptation. For example, face detection has been reported to occur within 100 ms of a face appearing (Crouzet, Kirchner, & Thorpe, 2010). The optimal stimulus to evoke this type of fast orientation towards faces is thought to consist of the characteristic contrast pattern of the face, three dark areas corresponding to the eye sockets and mouth surrounded by a lighter background (Tomalski, Csibra, & Johnson, 2009), which also generates preferential orienting in newborns (Farroni et al., 2005) and is hypothesized to be supported by a subcortical pathway involving the superior colliculus, pulvinar and amygdala (Johnson, 2005).

Eyes are a major conveyer of communicative signals. Human eyes are unique among primates, being horizontally elongated and possessing the largest ratio of exposed sclera within primates (Kobayashi & Kohshima, 2001). These two features are thought to have evolved in order to facilitate the detection of another’s gaze direction (Emery, 2000), enhancing the communicative value of gaze perception. Direct gaze is a relevant social cue signalling attention and/or intention toward oneself (Frischen, Bayliss, & Tipper, 2007; Senju & Johnson, 2009). It is preferentially detected by newborns (Farroni, Csibra, Simion, & Johnson, 2002) and modulates several concurrent tasks including emotion discrimination (Adams & Kleck, 2005) and identity encoding and retrieval (Conty & Grèzes, 2012; Hood, Macrae, Cole-Davies, & Dias, 2003). Furthermore, direct gaze is a particularly salient visual feature being detected faster than averted gaze in visual search tasks, the so-called “stare-in-the-crowd” effect (Doi & Shinohara, 2013; Senju, Hasegawa, & Tojo, 2005; Von Grünau & Anston, 1995). To account for the neural mechanisms underlying preferential processing of direct gaze, Senju and Johnson (2009) proposed a fast-track modulator model, which hypothesizes that direct gaze is initially detected by a subcortical pathway, which then subsequently modulates the cortical processing of social signals. Enhanced activation of amygdala for direct gaze detection was shown in a patient with total cortical blindness (Burra et al., 2013), which supports the claim that the subcortical pathway is sufficient to detect direct gaze.

One of the key predictions of the first-track modulator model is that detection of direct gaze facilitates express saccades, which are thought to rely on the superior colliculus (Schiller, Sandell, Maunsel, 1987). More specifically, it is predicted that rapid orienting to faces, as reported in previous studies (e.g. Crouzet & Thorpe, 2010), depends on direct gaze within the target face. To date, no study has directly tested this prediction [but see (Conty, Tijus, Hugueville, Coelho, & George, 2006; Doi and Shinohara, 2013, Palanica and Itier, 2011, Senju et al., 2005; Senju, Kikuchi, Hasegawa, Tojo, & Osanai, 2008; Von Grünau & Anston, 1995) for studies analysing direct gaze saliency with visual search tasks].

Here, we used a rapid orienting gap task to investigate the role of eye gaze in fast face detection. Rapid orienting tasks allow for saccadic responses as fast as 110 ms following the presentation of a face (Crouzet et al., 2010), and therefore should tap into any specialised processes for fast face detection. Moreover, the presence of a gap between the offset of fixation stimuli and the onset of target display has been shown to elicit express saccades (Fischer & Ramsperger, 1984). Express saccades rely on the superior colliculus (SC), being abolished in cases of SC lesion (Schiller et al., 1987). Targets’ visual properties have been shown to modulate express saccades, with concomitant modulation of SC activation (Bell, Meredith, Van Opstal, & Munoz, 2006; Marino, Levy, & Munoz, 2015). Significant differences between the detection of direct and averted gaze in this type of fast response would indicate a very fast processing of direct gaze.

Furthermore, significant differences between gaze directions were analysed at the cortical level, through the use of EEG. Saccade related Potentials [Presaccadic Positivity (PSP), the Spike Potential (SP) and the Lambda Wave] were analysed to examine the neural underpinnings of this fast detection and orienting. Compared to the typically used event related Potentials (ERPs) time-locked to stimulus onset, these components that are time locked to the saccade allow us to analyse the timecourse of cortical processing directly linked to fast face orienting. Furthermore the Lambda wave is structurally similar to fixation event-related potentials (fERPs, Dimigen, Kliegl, & Sommer, 2012; Kaunitz et al., 2014), possibly being closely linked with the visually evoked P100, N170, and P200. Faster detection of faces with direct gaze is expected with significant differences between gaze directions observed cortically in saccade locked ERPs during and after the onset of the saccade. Given the proposed role of subcortical structures for a faster detection of direct gaze, we do not expect differences between gaze directions, has measured cortically through the use of EEG, before saccade onset. We also explored a putative left visual field (LVF) bias for this effect since a better discrimination of gaze direction has been observed in the LVF (Ricciardelli, Ro, & Driver, 2002), as well as a more prominent effect of direct gaze (Palanica & Itier, 2011).

## 2 Materials and methods

### 2.1 Participants

Fifteen right handed volunteers (11 female, age range 24–48 years, 31.87 ± 9.69 years) participated in the experiment. All participants reported normal or corrected to normal vision and received payment or course credits. Written informed consent was obtained from all participants. The study was approved by the ethical committee of the Department of Psychological Sciences, Birkbeck, University of London.

### 2.2 Stimuli and procedure

**Fig. 1:**
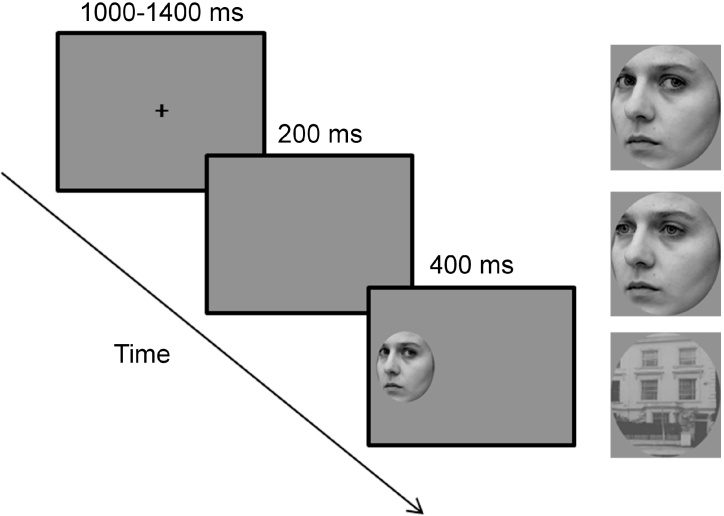
Experiment design and stimuli.

EEG was recorded while participants were asked to rapidly orient to targets that could be faces or buildings presented peripherally. Participants sat comfortably in an electrically shielded and soundproofed room at a fixed distance of 60 cm from the computer screen through the use of a chin rest. The experiment consisted of 6 blocks with 96 trials each. Buildings and faces were shown in equal numbers, with the latter balanced between gaze conditions. Each trial started with a fixation cross presented in the centre of the screen for 1000–1400 ms (randomly jittered), after which it disappeared leaving a gap of 200 ms before the stimulus onset (Fig. 1). The use of this gap paradigm allows for faster saccadic initiation (Fischer & Weber, 1993). Stimuli were then presented peripherally to one visual hemifield for 400 ms. Participants were instructed to fixate the centre of the screen until target appearance and then to saccade to the target stimulus as rapidly as possible.

Face stimuli were 12 greyscale digitized photographs of faces in neutral expressions and displaying a deviated head position with a rotation of 30° counterbalanced between right and left directions (George, Driver, & Dolan, 2001). Deviated head positions were used to avoid low level confounds, such as facial symmetry. Gaze condition was manipulated between direct and averted gaze (30°) counterbalanced between right and left gaze direction. Stimuli were cropped excluding hair and other non-facial cues. Twelve grayscale digitized photographs of buildings were also used as a control condition. Face and buildings’ stimuli were equated in mean luminance and contrast using the SHINE toolbox (Willenbockel et al., 2010). All images subtended 7.6° × 9.5° degrees of visual angle and were shown peripherally, 9.1° to the right or to the left of the fixation cross. Stimuli were presented using E-Prime software (Psychology Software Tools, Pittsburgh, PA).

### 2.3 ERP recording and data analyses

**Fig. 2:**
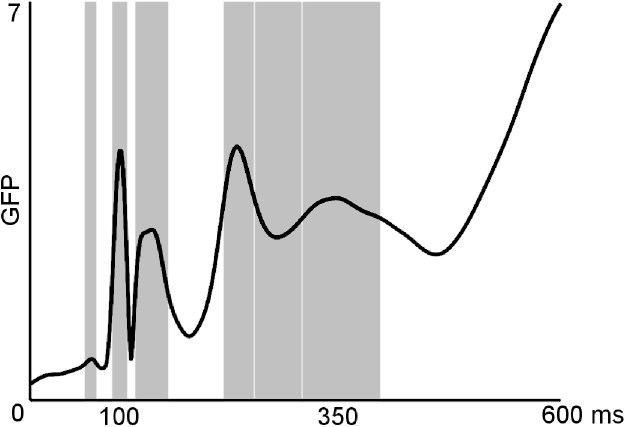
Global Field Power. Time windows analysed are in grey.

EEG was continuously recorded from 60 Ag-AgCl electrodes placed on a fitted cap (EASYCAP) according to the international 10/10 system. EOG electrodes were used to monitor eye-movements, with two electrodes placed on the canthi of right and left eye to detect horizontal movements, and one electrode placed below one eye to monitor vertical movements. Data was acquired at a sampling rate of a 1000 Hz. Electrode impedance was kept below 10 kΩ. EEG data was online referenced to the mastoids and offline re-referenced to an average reference. Data analysis was performed via the Matlab toolbox EEGLAB (Delorme & Makeig, 2004). Recordings were band pass filtered between 0.1 and 40 Hz, initially epoched (−100 ms to 1000 ms around target stimulus onset) and baseline corrected using the 100 ms prior to stimuli onset. Target locked epochs were used to calculate saccadic reaction time (SRT). This was defined as the time from the target stimulus onset to the execution of a correct saccade towards the target’s hemifield. Given the large number of express saccades occurring under 130 ms, which introduce considerable eye-movement artefacts, we do not analyse target locked ERPs here (though their analysis can be found in the Supplementary material, Section 1, and shows smaller amplitudes for direct than averted gaze in P100 in the left hemisphere and right hemifield and larger amplitudes for faces than buildings in the N170). Saccade onsets were automatically identified using the difference between the two horizontal EOG channels, as the beginning of a monotonic slope with more than 1 μV/ms in either direction lasting at least 20 ms (Csibra, Tucker, Volein, & Johnson, 2000). Trials with saccades beginning before 80 ms were excluded from further analysis as anticipatory responses. Trials with saccades starting after 500 ms were also rejected. Trials with small corrective saccades were kept while trials which included a switch of gaze direction occurring before 500 ms (i.e. the participant made an initial saccade in one direction followed by a second saccade to the opposite direction), were only kept for the behavioural analysis to obtain a measure of accuracy but excluded from the subsequent ERP analysis due to the associated double saccade response. Saccade locked epochs were created around saccadic onset (−100 ms to 500 ms), and were visually inspected to reject trials with artefacts. An average of 20.24% of trials was rejected per participant, including removal of trials with artefacts (16.02% including trials with a second saccade) and with incorrect saccades (4.02%). Saccade locked potentials consisted of the PSP, SP and the Lambda wave. Global field power (GFP, Skrandies, 1990) was used to select the time windows to analyse each of these components (Fig. 2). GFP uses the voltage at all electrodes for each sample of time to compute their spatial standard deviation, providing a single measure in which a larger signal standard deviation corresponds to more signal strength. Time windows were created considering around half the maximum GFP value of each component as the beginning and end of the time window. For the third component of the Lambda wave, given that it was a dip in the GFP, the time window was delimited by the boundaries of its previous and following components.

PSP amplitude was analysed in the time window of 38 to 25 ms prior to the saccade onset. The SP was defined as a sharp positivity peaking around saccade onset and was analysed between −7 ms to 10 ms around it. The four components of the Lambda wave were defined as an initial negativity followed by a similar structure as the typical P1, N170 and P200 occurring after saccade offset, and were respectively analysed in four time windows 19–56 ms, 119–153 ms, 154–207 ms, 208–296 ms after saccade onset. All components were analysed in parieto-occipital regions (P7/P8, P5/P6, PO7/PO8, see Supplementary materials, Fig. S4 for parieto-occipital waveforms), areas which are commonly accepted to best capture these components (Jagla, Jergelová, & Riecanský, 2007; Richards, 2013). Statistical analyses of the mean amplitudes in each time window were carried out using a three-way ANOVA with hemisphere (right and left), visual hemifield of target (right and left visual presentation) and condition (buildings, averted and direct gaze) as within-subject factors.

As in previous studies using a gap paradigm (Fischer & Weber, 1993), we found a bi-modal distribution of saccades in several of the participants (see Supplementary materials, Fig. S5) indicating that there are two populations of saccades, express saccades and standard saccades. Considering previous literature, express saccades were defined as saccades occurring between 80 and 130 ms after stimulus onset (Kikuchi et al., 2011, Knox and Wolohan, 2015, Ozyurt and Greenlee, 2011; Van der Stigchel, Imants, & Ridderinkhof, 2011). Individual thresholds for express saccades, were not used given the difficulty to identify them in some of the participants.

SRT and accuracy of express saccades, as well as all the recorded saccades, were analysed with a two-way ANOVA with visual hemifield and condition as within-subject factors. When appropriate, post hoc planned comparisons were performed using two-tailed paired *t*-tests. Violations of sphericity were corrected with the Greenhouse-Geisser correction.

## 3 Results

### 3.1 Behavioural results

#### 3.1.1 Express saccades

**Fig. 3:**
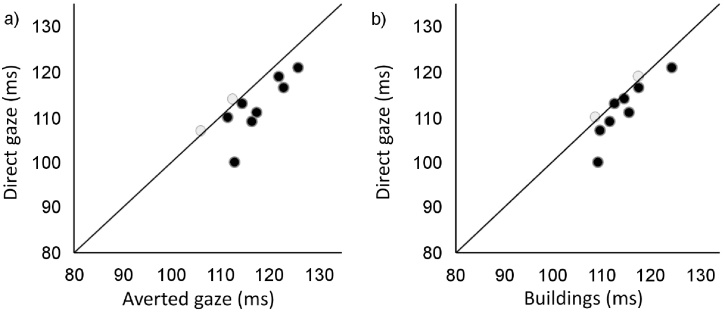
Express saccades reaction time to orientation in the left visual to (a) faces in averted and direct gaze and (b) faces in direct gaze and buildings.

The percentage of express saccades varied across participants ranging from 4.86% to 77.6% (M = 33.73%) with no significant differences between conditions (F(2, 28) = 1.51, *p *= 0.238), hemifields (F(1, 14) = 1.10, *p *= 0.312) or an interaction between these factors (F(2, 28) = 1.59, *p *= 0.222). Accuracy of express saccades (i.e. saccades occurring between 80 and 130 ms) was at ceiling level (98.8%). To analyse participants’ median express saccades’ SRT, only participants with a minimum of 10 trials with accurate express saccades per condition were included, leading to the exclusion of five participants. In the remaining 10 participants an interaction between condition and hemifield was found (F(2, 18) = 8.56, *p *= 0.002). This effect was driven by the conditions on the left hemifield (F(2, 18) = 6.97, *p *= 0.006), in the absence of any effect on the right hemifield (F(2, 18) = 0.05, *p *= 0.948). Post-hoc comparisons showed faster saccadic reaction times on the left hemifield for direct gaze (M = 112.05 ms, Fig. 3, Table 1) compared with averted gaze (M = 116.25 ms, *p *= 0.015) and buildings (M = 114.55 ms, *p *= 0.035), in the absence of a significant difference between averted gaze and buildings (*p *= 0.102).

#### 3.1.2 All saccades

Analysis of all saccades, including express saccades was performed in all participants to match the analysed electrophysiological data. Accuracy on the saccade task had a mean of 84.86% (range: 63.37% to 97.74%) with no effect of condition (F(2, 28) = 0.44, *p *= 0.647), hemifield (F(1, 14) = 0.05, *p *= 0.819) or an interaction between these factors (F(2, 28) = 1.04, *p *= 0.367). Similarly when analysing the participants median SRT, no effect of condition (F(2, 28) = 0.47, *p *= 0.628), hemifield (F(1, 14) = 1.98, *p *= 0.181) or interaction (F(2, 28) = 0.25, *p *= 0.780) was observed.

### 3.2 Electrophysiological data

**Fig. 4:**
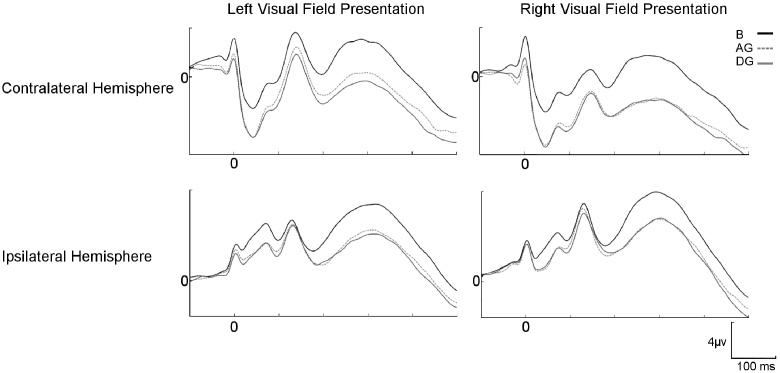
ERP waveforms, with zero as the onset of the saccade, in ipsi and contralateral parieto-occipital electrodes (P7/P8, P5/P6, PO7/PO8) to left and right visual field stimuli presentation. B—buildings; AG—averted gaze; DG—direct gaze.

An ERP analysis, was performed over all trials with correct saccades in all participants. All the saccade-locked potentials showed a significant interaction between hemisphere, hemifield and condition (PSP, F(2, 28) = 13.38, *p* < 0.001; SP, F(2, 28) = 19.11, *p* < 0.001; the first negativity (F(2, 28) = 19.10, *p* < 0.001), first positivity (F(1.2, 17.33) = 18.65, *p* < 0.001), second negativity F(1.45, 20.35) = 18.86, *p* < 0.001) and second positivity (F(2, 28) = 14.75, *p* < 0.001) of the Lambda wave. Follow-up analyses revealed that the PSP and SP showed an interaction between hemifield of presentation and stimulus condition in both right (PSP: F(2, 28) = 5.79, *p *= 0.008) and left (F(2, 28) = 5.98, *p *= 0.007; SP: right: F(2, 28) = 8.72, *p *= 0.001; left: F(2, 28) = 8.47, *p *= 0.001) hemisphere electrodes. The four components of the Lambda wave also showed significant hemifield x condition interaction in both hemispheres (right: F(2, 28) = 3.78, *p *= 0.035; F(2, 28) = 4.46, *p *= 0.021; F(2, 28) = 8.83, *p *= 0.001; F(2, 28) = 4.92, *p *= 0.015; left: F(2, 28) = 11.77, *p* < 0.001; F(1.34, 18.77) = 16.70, *p* < 0.001; F(2, 28) = 15.45, *p* < 0.001; F(2, 28) = 14.24, *p* < 0.001). Note that as no significant differences were found between faces with right and left averted gaze on all the components analysed this factor was collapsed. Given that no significant effects of gaze were observed in any component in the right hemifield or in the left hemisphere (see Fig. 4 for the ERP waveforms) the following sections will focus on the effects observed in the left hemifield and right hemisphere (see Supplementary material for the remaining combination of hemifields and hemispheres).

#### 3.2.1 PSP

Significant differences between conditions were found for left hemifield presentation on the right hemisphere electrodes (F(2, 28) = 6.14, *p = *.006). Pairwise comparisons (LSD) revealed a larger PSP amplitude for buildings (M = 2.11 μV) than for faces with direct gaze (M = 0.69 μV, p = 0.007) and marginally so than for faces with averted gaze (M = 1.18 μV, p = 0.057). No significant differences were found between gaze directions (p = 0.146).

#### 3.2.2 SP

Significant differences were observed (F(1.20, 16.83) = 15.16, p = 0.001) with a smaller positive peak for averted (M = 1.59 μV, p = 0.004) and direct gaze (M = 1.16, p = 0.001) than for buildings (M = 3.41 μV). More importantly differences between gaze directions were significant (p = 0.041), with a smaller SP for direct gaze.

Since this component has been associated with the corneo-retinal dipole and extra-ocular muscular signals (Thickbroom and Mastaglia, 1985), an analysis of the two horizontal EOG channels was also performed, but no effect of condition was significant (F(2, 28) = 0.65, p = 0.532).

#### 3.2.3 Lambda wave

Four components of the lambda wave were distinguishable, an initial negative component peaking around 18–46 ms (L1), a positive component peaking at 129–147 ms after saccade onset (L2), a negative component with a peak at 176–199 ms (L3) and a positive broad component with a peak between 273 and 318 ms (L4). differences between conditions were found (L1: F(1.40, 19.64) = 38.98, *p* < 0.001; L2: F(1.28, 17.91) = 14.17, *p* = 0.001; L3: F(1.39, 19.46) = 20.40, *p* < 0.001; L4: F(1.32, 18.41) = 49.36, *p* < 0.001), with smaller amplitudes for averted (L1: M = −5.67 μV, *p* < 0.001; L2: M = 2.32 μV, *p* = .003; L3: M = −0.69 μV, *p *= 0.001; L4: M = −0.45 μV, *p* < 0.001) and direct gaze (L1: M = −5.73 μV, *p* < 0.001; L2: M = 1.53 μV, *p *= 0.001; L3: M = −1.45, *p* < 0.001; L4: M = −1.34 μV, *p* < 0.001) than for buildings (L1: M = −2.68 μV; L2: M = 4.03 μV; L3: M = 1.35 μV; L4: M = 3.04 μV) in the four components of the lambda complex. Again, differences between direct and averted gaze were significant with smaller amplitudes for direct than averted gaze in the last three components (p=0.013; p=0.016; p=0.004) but not on the first (p=0.802).

#### 3.2.4 Saccade-locked ERPs for trials with express saccades

Given that behavioural findings were observed only for express saccades, an additional analysis was performed on the ERP data to confirm that a similar pattern was observed for trials in which express saccades were observed (see Fig. S6 in the Supplementary material for the waveforms). This analysis was performed for seven participants with more than 15 trials with express saccades per condition (mean number of express saccade trials per condition in these participants was 52.2 ± 16.2). We focused on the critical condition of left hemifield presentation and right hemisphere activation, the only hemifield/hemisphere combination where significant differences between gaze directions were observed in the full data set. The same pattern of results was observed for trials with express saccades, with a main effect of condition occurring in all components (SP, F(2, 12) = 5.91, p = 0.016; L1, F(2, 12) = 7.22, p = 0.009; L2, F(1.12, 6.74) = 5.76, p = .046; L3, F(2, 12) = 5.79, p = 0.017; L4, F(1.16, 6.99) = 16.24, p = 0.004) except PSP (F(2, 12) = 0.91, p = 0.429), where originally no effect of gaze was found. The effects of gaze were similar in trials with express saccades, with smaller/more negative amplitudes for direct (SP, M = 4.46; L2, M = 1.77; L4, M = −0.93) than averted gaze (SP, M = 5.11; L2, M = 2.66; L4, M = −0.05) in the SP (p = 0.028), and in the two positive components of the Lambda Wave, L2 (p = 0.024), and L4 (p = 0.031). The only component in which this effect was not replicated was the second negativity of the Lambda Wave (L3, p = 0.234), where nonetheless the same numerical trend was observed with more negative amplitudes for direct (M = −0.99) than averted gaze (M = −0.39). A similar pattern of differences between faces and buildings was observed, with the exception of the PSP, where no significant difference between faces and buildings was observed (*p* = 0.823 and *p* = 0.373 for averted and direct gaze respectively) and the SP and L2 where only direct gaze significantly differed from buildings. This replicates the behavioural findings in the SP, where faces with direct gaze significantly differed from buildings (M = 5.21, p = 0.003) but faces with averted gaze did not (*p* = 0.756). In the Lambda Wave components, a significant or marginally significant difference, in the case of L2, was observed, with faces with averted (L1, p = 0.015; L2, p = 0.095; L3, p = 0.042; L4, p = 0.009) and direct gaze (L1, p = 0.035; L2, p = 0.041; L3, p = 0.048; L4, p = 0.005) showing smaller/more negative amplitudes than buildings.

## 4 Discussion

This is the first study to show that faster express saccades to faces occur only for faces with direct gaze in the left visual hemifield. Note that the latency of express saccades for faces with averted gaze and for buildings did not differ significantly, which suggests that the rapid orienting to faces when compared with other objects (Crouzet et al., 2010) might depend on the perception of direct gaze. In addition, such a rapid orienting to direct gaze was only observed in the left visual hemifield, consistent with a left visual hemifield bias previously found for rapid face detection (Crouzet et al., 2010). This finding also highlights the key role of left visual hemifield in gaze processing (Palanica and Itier, 2011, Ricciardelli et al., 2002). Such a rapid orienting to faces with direct gaze would potentially contribute to the immediate detection of another person's attention to oneself, and the timely response to initiate social communication.

This study is also the first to report the modulation of early saccade-locked ERP components by direct gaze compared to averted gaze, particularly when participants showed express saccades. Differential ERP components between gaze directions were initially observed during saccade onset (i.e. SP), which occurred in average around 140 ms for all saccades and 114 ms for express saccades after the stimulus onset, over occipito-parietal regions of the right hemisphere, suggesting rapid processing of direct gaze. It is unlikely that the modulations of ERPs by direct gaze seen for left hemifield stimuli is attributed to differential eye movements of the participants, since no significant effect of eye gaze condition was observed in the electrooculography signals recording the eye movements themselves.

The earliest effect of gaze direction on the ERP components was found in the amplitude of the SP, which peaked around saccade onset, and showed reduced amplitude for saccades to direct compared to averted gaze faces. This potential can often be observed immediately preceding saccades and has been attributed to saccade planning, peaking over parietal electrodes [(Csibra et al., 2000, Richards, 2013) but see (Carl, Açık, König, Engel, & Hipp, 2012; Thickbroom & Mastaglia, 1985)]. Smaller amplitudes in the SP have previously been associated with better efficiency (Jagla et al., 2007). The average latency of the SP, linked with mean SRT of 140 ms on all saccades and 114 ms in express saccades, was shorter than the component most commonly associated with gaze processing (N170, Conty, N’Diaye, Tijus, & George, 2007; Itier, Alain, Kovacevic, & McIntosh, 2007; Puce, Smith, & Allison, 2000). This effect cannot be explained by the influence of target-locked ERP, because it did not differentiate between direct and averted gaze in the right hemisphere for left hemifield stimuli presentation, where we observed gaze effects in saccade-locked ERP. Earlier effects of direct gaze in ERP components have seldomly been found, and mostly in tasks that draw attention towards the eyes (Berchio et al., 2016, Klucharev and Sams, 2004, Schmitz et al., 2012). In Klucharev and Sams (2004) study participants were asked to attend to emotional and neutral faces with direct or averted gaze and to indicate whenever there was a repetition of gaze direction. A larger positivity was found at parieto-occipital areas for direct gaze at 85 ms.

Direct gaze observed in the left visual hemifield also generated more negative amplitudes than averted gaze in the three lambda wave components that structurally resemble the P100, N170 and P200. The lambda wave has been associated with fixation related components (Dimigen et al., 2012, Kaunitz et al., 2014), with similar dipoles between the first lambda component and P100 (Kazai & Yagi, 2003). Interestingly in the present study, the differences between direct and averted gaze found in the second negativity of the Lambda complex was not observed in the target locked N170. The majority of previous studies using static images in adult typical populations were unable to find any differences between gaze directions in these components (Grice et al., 2005, Klucharev and Sams, 2004; Nomi, Frances, Nguyen, Bastidas, & Troup, 2013; Taylor, George, & Ducorps, 2001; Taylor, Itier, Allison, & Edmonds, 2001; Yokoyama, Noguchi, & Kita, 2013), with two finding larger N170 for averted gaze (Itier et al., 2007, Watanabe et al., 2002). A similar difference in N170 was observed in Conty et al. (2007), where gaze dynamically changed from a neutral position to direct or averted gaze. This might suggest that the establishment of eye contact either by the observer or by the stimuli is crucial for an enhanced processing of direct gaze.

One caveat of the present study is that by using only EOG, we were not able to analyse the landing point of the saccade. Future studies using eye-tracking permit the analysis of the precision of the saccades in each condition as well as the area of the face each saccade landed on which could be an important factor in driving the Lambda Wave.

It is also of note that we did not observe any differences between gaze conditions in the components prior to the saccadic onset, which was also the case for express saccades. These results are consistent with the prediction derived from the fast-track modulator model, and could support the claim that rapid detection of direct gaze involves a subcortical pathway (Senju & Johnson, 2009), as the activity of subcortical structures is generally not directly detected by the surface-recorded ERP. The observed advantage of direct gaze, as predicted, in a task of rapid orienting to faces, could suggest the involvement of the SC given the importance of this structure for the execution of express saccades.

Further studies are needed to understand the neural underpinnings of this effect, with complementary methodologies to directly assess the contribution of the subcortical pathway, such as fMRI and/or neuropsychological studies with patients with focal lesion in the subcortical pathway.

## Figures and Tables

**Table 1:** Behavioural results.

	Left Hemifield			Right Hemifield		
	Buildings	AG	DG	Buildings	AG	DG
Mean SRT (ms)	140.77 ± 23.41	140.30 ± 23.28	140.77 ± 25.89	144.90 ± 26.06	144.70 ± 21.98	146.43 ± 23.22
Mean express saccades SRT (ms; N = 10)	114.55 ± 4.96	116.25 ± 6.06	112.05 ± 6.13	115.85 ± 3.33	116.15 ± 5.28	116.15 ± 4.15
% of accuracy	84.91 ± 12.61	84.81 ± 14.32	85.74 ± 13.17	84.26 ± 14.37	86.02 ± 13.81	83.98 ± 14.57
% of correct express saccades	34.54 ± 25.98	36.57 ± 24.89	34.91 ± 26.53	33.56 ± 23.39	31.94 ± 23.68	30.19 ± 23.25
