# Supplementary material for: Direct gaze facilitates rapid orienting to faces: Evidence from express saccades and saccadic potentials
Source: Biol Psychol. 2016 Dec;121(Pt A):84–90. doi: 10.1016/j.biopsycho.2016.10.003 (PMC5157993; doi:10.1016/j.biopsycho.2016.10.003)
Supplement: Supplementary file 1 [file mmc1.pdf]

1. Target locked analysis – Methods and results

1.1. Data analysis method of target locked ERPs

Target locked ERPs were analysed in a similar way as saccade locked ERPs for the key components relevant to the main findings. The epochs used for this analysis were centred around stimulus onset (-100 ms to 500 ms). For comparability, the trials used for this analysis were the same as those used for the analysis of all saccades, which excluded trials with saccades beginning before 80 ms and after 500 ms. Incorrect trials or trials which included a switch of gaze direction occurring before 500 ms, were also excluded from the subsequent ERP analysis due to the associated double saccade response. As with the main analysis global field power (GFP, Skrandies, 1990) was used to identify the time windows to analyse the selected components, P100 and N170 (Figure S1). The beginning and end of each time window was set around half the maximum GFP value of each component. Consequently, P100 and N170 mean amplitudes were respectively analysed between 99 and 130 ms and 172 and 205 ms. All components were analysed in the same parieto-occipital regions as the main analysis (P7/P8, P5/P6, PO7/PO8) to allow for comparison (Figure S2 for topographical maps).

Statistical analyses of the mean amplitudes in each time window were carried out using a three-way ANOVA with hemisphere (right and left), visual hemifield of target (right and left visual presentation) and condition (buildings, averted and direct gaze) as within-participant factors. When appropriate, post hoc planned comparisons were performed using two-tailed paired t-tests. Violations of sphericity were corrected with the Greenhouse-Geisser correction.

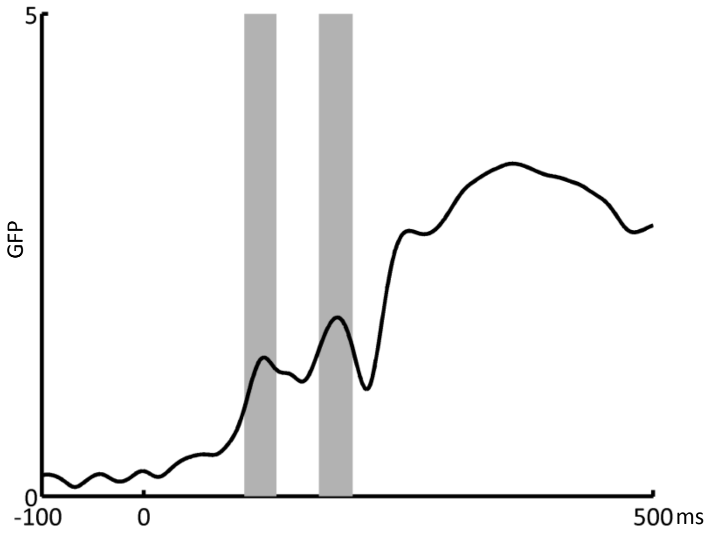

Figure S1 – Global Field Power for target locked saccades. Time windows analysed for P100 and N170 are in grey.

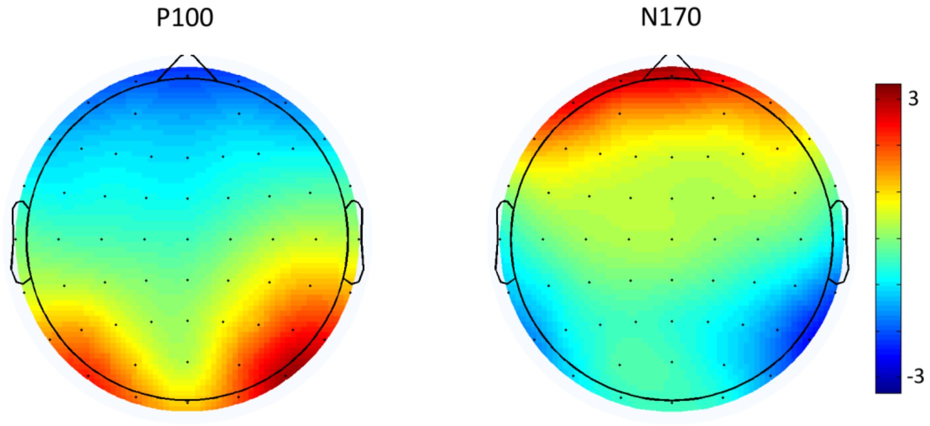

Figure S2 Topographies of target locked P100 and N170.

1.2. Results for Target Locked ERPs

1.2.1. P100

A three-way interaction between hemisphere, hemifield and condition was significant ($F(2,28)= 9.13$, $p=.001$), which was driven by an interaction between hemifield and condition on the left hemisphere ($F(2,28)= 8.78$, $p=.001$, see Figure S3 for ERP waveforms). Differences between conditions occurred only in the contralateral hemifield (right hemifield; $F(2,28)= 6.58$, $p=.005$), where faces with averted gaze had a smaller ($M=1.60$) amplitude than faces

with direct gaze (M=2.55; p=.009) or buildings (M=2.76; p=.010). This difference was unexpected, and different from the combination of hemifield and hemisphere which yielded the main results.

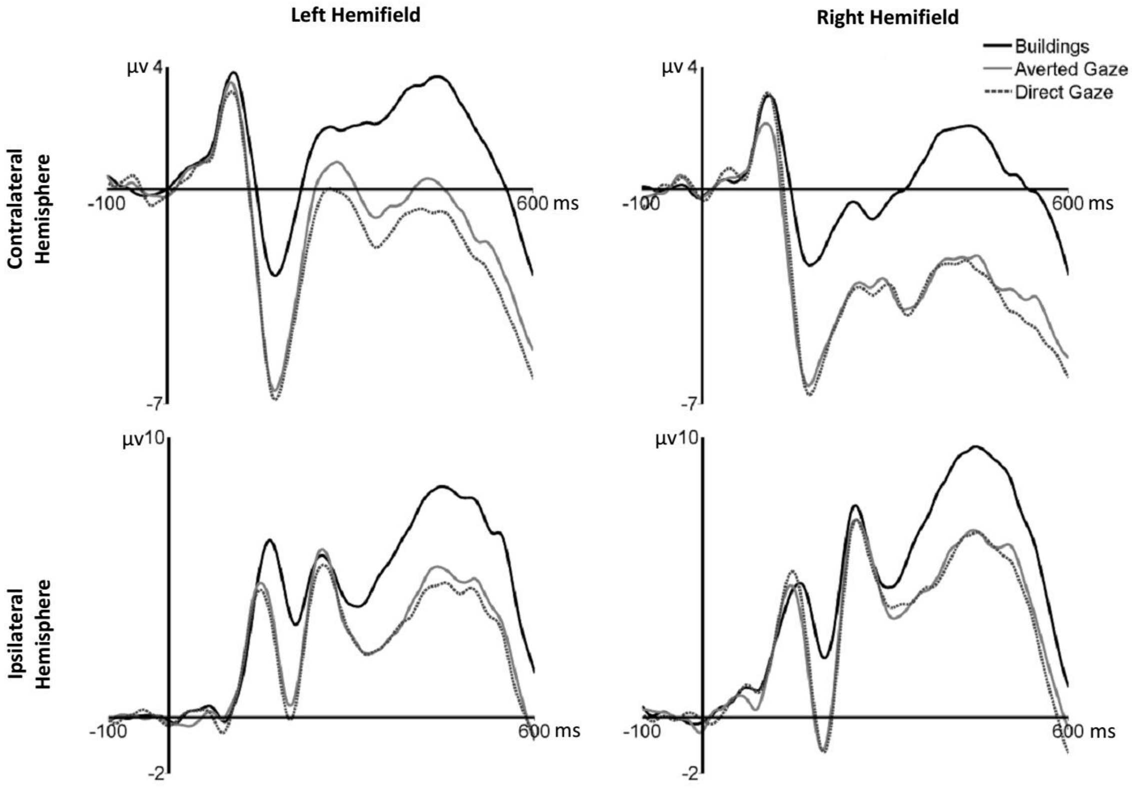

Figure S3 ERP waveforms for target locked epochs, in ipsi and contralateral parieto-occipital electrodes (P7/P8, P5/P6, PO7/PO8) to left and right visual field stimuli presentation.

1.2.2. N170

Main effects of hemifield (F(1,14)= 4.75, p=.047) and condition (F(1.11,28)= 55.33, p<.001) were significant, which was driven by larger amplitudes in the right hemifield (M=-1.92) than the left (M=-1.20; p=.047), and to faces with direct (M=-2.84, p<.001) and averted gaze (M=-2.61, p<.001) than buildings (M=-.78). No difference was significant between gaze directions (p=.102). A three way interaction between hemisphere, hemifield and condition was not significant either (F(1.27,17.79)=.72, p=.439). This overall face specific effect, which

occurred independently of hemisphere and hemifield, could partially account for the differences observed in saccade-locked components. Nonetheless in express saccades where the spike potential precedes the N170, a significant difference between faces with direct gaze and buildings was still observed, showing that these effects cannot be fully justified by target locked differences.

2. Saccade Locked ERP analyses for additional combination of hemifield and hemisphere

2.1. Left hemifield presentation in the left hemisphere

No differences between conditions were significant for left hemifield presentation on the left hemisphere for the PSP ($F(2, 28) = .86$, $p=.433$), SP ($F(2,28)=2.81$, $p=.077$) and second Lambda component ($F(2,28)=1.40$, $p=.262$).

Differences between conditions in left hemifield were significant in left hemisphere regions in the first, third and fourth Lambda components ($F(1.44,20.17)= 17.93$, $p<.001$; $F(1.43,20.01)=6.43$, $p=.012$; $F(1.19,16.70)=20.29$, $p<.001$). This was driven by differences between buildings (M=4.67 µV; M=3.67 µV; M=7.07 µV) and faces with averted (M=2.74 µV, $p=.002$; M=2.25 µV, $p=.014$; M=3.97 µV, $p=.001$) and direct gaze (M=2.40 µV, $p<.001$; M=2.27 µV, $p=.021$; M=3.59, $p<.001$), with no differences between gaze conditions ($p=.225$; $p=.932$; $p=.162$).

2.1.1. Right hemifield presentation

Presaccadic positivity (PSP). Differences in PSP between conditions were significant for right hemifield presentation on the left hemisphere ($F(2, 28) = 7.61$, $p=.002$) but not on the right ($F(2, 28) = 1.26$, $p=.299$). A significantly larger PSP amplitude was found for

buildings (M=1.10 µV) than for faces in direct gaze (M=.32 µV, p=.027) and averted gaze (M=-.12 µV, p=.001). No differences were significant between gaze directions (p=.222).

Spike potential (SP). Differences between conditions for right hemifield presentation were only significant in left hemisphere electrodes ($F(1.33,18.63)=15.04$, $p<.001$). Pairwise comparisons revealed again a smaller SP for averted gaze (M=.73 µV, p<.001) and direct gaze (M=1.29 µV, p=.005) than buildings (M=3.44 µV). No significant difference was found between gaze directions (p=.074).

As discussed above for left hemi-field presentation, an analysis of visual presentation by hemifield was conducted on the signals recorded over EOG electrodes. A significant effect was found for right hemifield presentation on the left hemisphere ($F(2,28)=4.07$, $p=.028$) but not on the right hemisphere ($F(2,28)=1.67$, $p=.207$). Differences on the left hemisphere were significant between buildings (M=-11.23 µV) and averted gaze (M=-12.33 µV, p=.021) with a marginal difference with direct gaze (M=-12.34 µV, p=.065). No difference was found between gaze directions (p=.985).

Lambda complex. Differences between conditions for right hemifield presentation in the four lambda wave components, were significant in left hemisphere regions (L1: $F(1.25,17.50)= 69.35$, $p<.001$; L2: L3: $F(1.10,15.35)= 15.67$, $p=.001$; $F(1.10,15.38)= 18.40$, $p<.001$; L4: $F(1.22,17.13)= 54.42$, $p<.001$). Again, a significantly smaller amplitude was found for averted gaze (L1: M=-6.26 µV, $p<.001$; L2: M=-2.31 µV, p=.001; L3: M=-3.28 µV, p<.001; L4: M=-2.75 µV, p<.001) and direct gaze (L1: M=-6.31 µV, p<.001; L2: M=-2.36 µV, p=.001; L3: M=-3.25 µV, p=.001; L4: M=-2.62 µV, p<.001) than buildings (L1: M=-2.83 µV; L2: M=.36 µV; L3: M=-.48 µV; L4: M=1.86 µV). Importantly, no significant differences were found between gaze directions (L1: p=.806; L2: p=.780; L3: p=.854; L4: p=.580).

Differences between conditions in the first, third and fourth Lambda components were also significant in right hemisphere regions (L1: F(2,28)=16.31, *p*<.001; L3: F(1.45,20.36)=4.75, *p*=.029; L4: F(1.37,19.14)= 32.74, *p*<.001). Pairwise comparisons revealed significantly smaller amplitudes for averted gaze (L1: M=1.31 µV, *p*<.001; L3: M=3.44 µV, p=.012; L4: M=5.57µV, p<.001) than for buildings (L1: M=3.29 µV; L3: M=4.72µV; L4: M=8.55 µV) in these three components of the lambda wave. Differences between buildings and direct gaze were significant in the first and fourth component (direct gaze: L1: M=1.51, p=.002; L4: M=5.49 µV, p<.001). Again, no significant differences were found between direct and averted gaze (L1: p=.514; L3: p=.144; L4: *p*=.769).

3. Other Supplementary figures

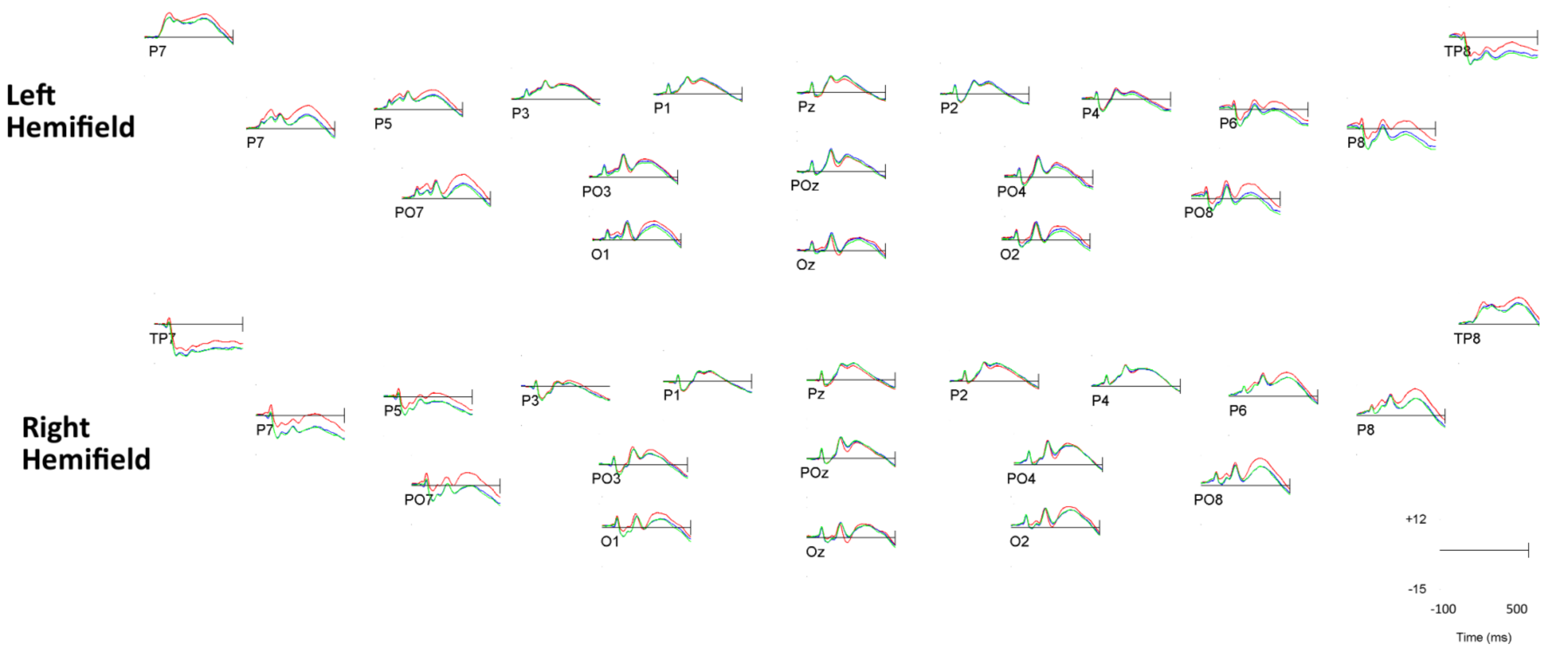

Figure S4 Saccade locked ERP waveforms over occipito-parietal areas. Red, buildings; Blue, averted gaze; Green, direct gaze.

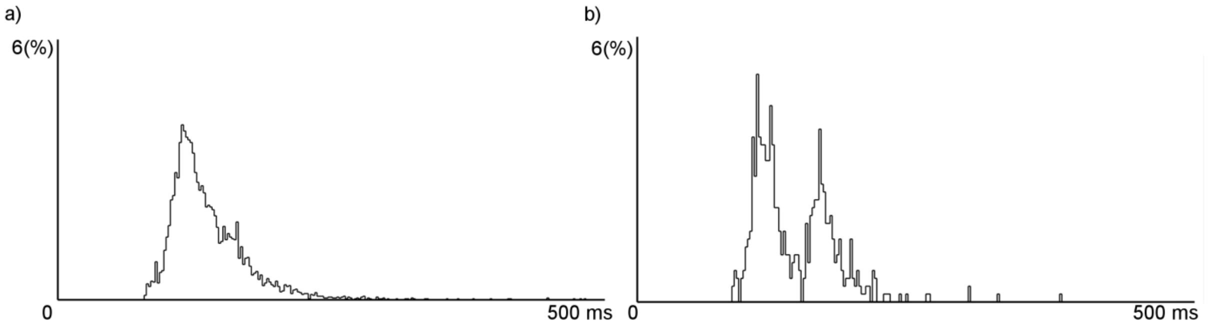

Figure S5 Distribution of saccadic reaction times on (a) the 10 participants that displayed more than 10 express saccades per condition and in (b) a single subject. Bin width: 2 ms. Y-axis, percentage of saccades; X-axis, Saccadic reaction time.

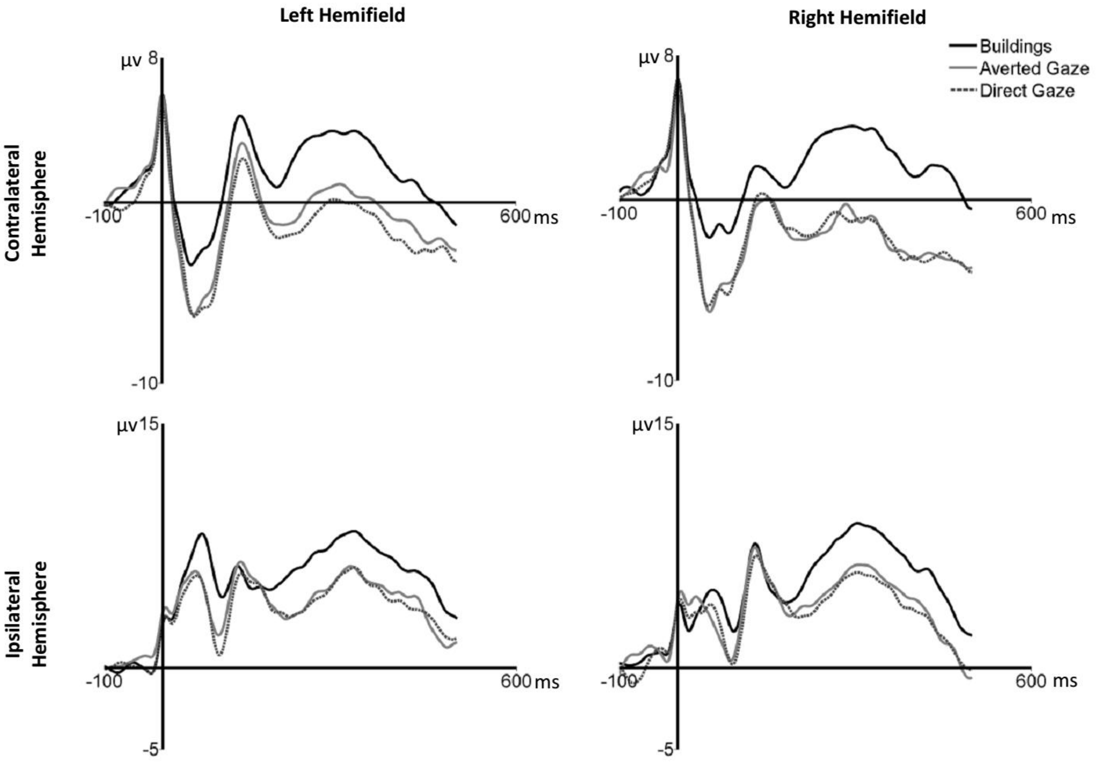

Figure S6 ERP waveforms for saccade locked epochs in express saccades, in ipsi and contralateral parieto-occipital electrodes (P7/P8, P5/P6, PO7/PO8) to left and right visual field stimuli presentation.
